# Geniposide Alleviates Isoproterenol-Induced Cardiac Fibrosis Partially via SIRT1 Activation *in vivo* and *in vitro*

**DOI:** 10.3389/fphar.2018.00854

**Published:** 2018-08-03

**Authors:** Ning Li, Heng Zhou, Zhen-Guo Ma, Jin-Xiu Zhu, Chen Liu, Peng Song, Chun-Yan Kong, Hai-Ming Wu, Wei Deng, Qi-Zhu Tang

**Affiliations:** ^1^Department of Cardiology, Renmin Hospital of Wuhan University, Wuhan, China; ^2^Cardiovascular Research Institute, Wuhan University, Wuhan, China; ^3^Hubei Key Laboratory of Cardiology, Wuhan, China

**Keywords:** SIRT1, Geniposide, cardiac fibrosis, stress, Smad3

## Abstract

**Objective:** Geniposide (GE) is a major component in the fruit of *Gardenia jasminoides* Ellis. Oxidative stress, endoplasmic reticulum (ER) stress, and canonical Smad3 pathway are implicated in the pathogenesis of cardiac fibrosis. We aim to investigate the protective roles of GE in isoproterenol (ISO)-induced cardiac fibrosis.

**Methods:** ISO was used to induce cardiac fibrosis in male C57BL/6 mice. GE and the EX-527 were given for 2 weeks to detect the effects of GE on cardiac fibrosis. Levels of oxidative stress, ER stress, and Smad3 were evaluated by real time-PCR, Western blots, immunohistochemistry staining, immunofluorescence staining, and assay kits.

**Results:** GE treatment alleviated cardiac dysfunction, fibrosis, and hypertrophy in mice response to ISO. Additionally, GE also suppressed the transformation of cardiac fibroblasts to myofibroblasts stimulated by transforming growth factor-β (TGF-β) *in vitro*. Mechanistically, GE inhibited the oxidative stress, ER stress, as well as Smad3 pathway activated by ISO or TGF-β. A selective antagonist of sirtuin 1 deacetylase (SIRT1), EX-527, partially counteracted the anti-fibrotic effect and weakened the inhibitory effect on the transformation of cardiac fibroblasts to myofibroblasts after the treatment of GE. Acetylated Smad3 (ac-Smad3), oxidative stress, as well as ER stress pathway were significantly enhanced after SIRT1 was blocked while phosphorylated Smad3 (P-Smad3) was not affected.

**Conclusion:** GE could combat cardiac fibrosis *in vivo* and *in vitro* by inhibiting oxidative stress, ER stress, and ac-Smad3 in a SIRT1-dependent manner and suppressing P-Samd3 pathway independent of SIRT1 activation. GE is expected to be a promising agent against cardiac fibrosis.

## Introduction

Cardiac fibrosis is a common pathological feature in many cardiovascular diseases, involving hypertension, myocardial infarction, dilated cardiomyopathy, and diabetes mellitus (Gyöngyösi et al., 2017). In the progression of cardiac fibrosis, excessive production and deposition of extracellular matrix (ECM) and collagen significantly increase ventricular stiffness and deteriorate diastolic function, which eventually give rise to heart failure (Travers et al., 2016). Currently, the precise mechanisms regulating the process of cardiac fibrosis remain incompletely understood, but emerging evidence supports a critical role of the transforming growth factor-β (TGF-β)/Smad signaling pathway in cardiac fibrosis. Activated TGF-β1 may recruit and activate the downstream serine kinases type I TGF-β receptor (Tβ RI) and type II TGF-β receptor (Tβ RII) to phosphorylate Smad2 and Smad3. Once Smad2 and Smad3 are phosphorylated, they form a Smad complex with Smad4 and enter the nucleus, inducing protein expression associated with cardiac fibrosis (Margadant and Sonnenberg, 2010). Meanwhile, Smad3 can be acetylated by p300/CBP at Lys-378 in the MH2 domain, which regulates Smad3 transcriptional activity and Smad3 DNA-binding activity in response to profibrotic response (Inoue et al., 2007; Li et al., 2010). Additionally, oxidative and endoplasmic reticulum (ER) stresses also possess close relationships with cardiac fibrosis. Cardiac oxidative stress not only enhances cardiac collagen synthesis and inhibits collagen degradation in perivascular/interstitial fibrosis in hypertensive rats (Zhao et al., 2008) but also provokes accumulation of ECM in the left ventricle (LV) of streptozotocin (STZ)-induced diabetic rats by activating several genes correlated with fibrosis, such as fibronectin, TGF-β1, and connective tissue growth factor (CTGF), in addition to activation of the NF-κB pathway (Aragno et al., 2008). In cardiac fibrosis, ER stress and the unfolded protein response (UPR) are significantly activated, accompanied by the upregulation of ER stress markers, including ATF4, peIF2α, and CHOP. Thus, suppression of oxidative stress, ER stresses, and the TGF-β/Smad signaling pathway is regarded as a promising therapy for cardiac fibrosis (Ayala et al., 2012; Kassan et al., 2012).

Sirtuin 1 deacetylase (SIRT1), the closest mammalian homolog of the yeast silent information regulator 2 protein, is a nicotinamide adenine dinucleotide NAD(+)-dependent deacetylase (Brachmann et al., 1995; Kaeberlein et al., 1999; Suzuki and Bartlett, 2014). SIRT1 may interfere with cardiac fibroblast activation by inhibiting the P-Smad3 pathway, eventually reducing cardiac fibrosis (Cappetta et al., 2015). SIRT1 also possesses an anti-apoptosis effect by inhibiting ER stress via the ATF6/CHOP, PERK/eIF2α, and IRE1α/JNK-mediated pathways (Cappetta et al., 2015). In a rodent study, both hepatic steatosis and insulin resistance could be reversed through SIRT1 overexpression, the mechanism of which may be explained by the inhibition of ER stress (Li et al., 2011). Resveratrol, an activator of SIRT1, could prevent generation of reactive oxygen species (ROS) in cardiomyocytes through SIRT1 and mitochondrial biogenesis signaling pathways (Li Y.G. et al., 2013). These studies indicate that in the heart, the Smad3 signaling pathway, ER stress, and oxidative stress can be regulated by SIRT1.

**FIGURE 1:**
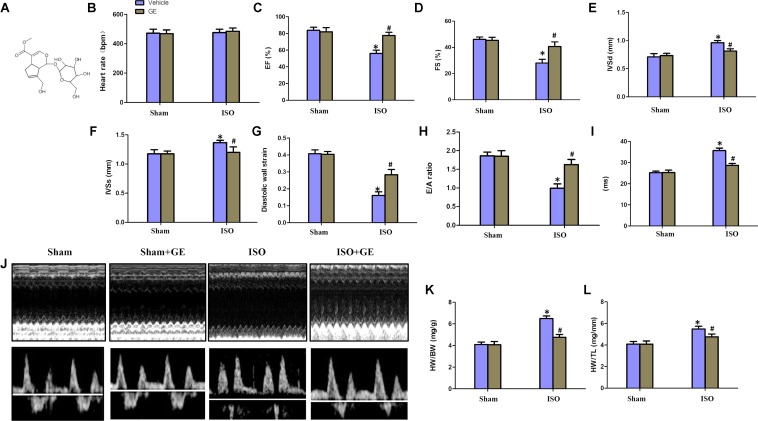
Geniposide (GE) prevented cardiac dysfunction in mice challenged with isoproterenol (ISO). **(A)** Chemical structure of GE. **(B–I)** Echocardiographic parameters including heart rate, EF, FS, interventricular septal thickness at diastole or systole (IVSd or IVSs), diastolic wall strain (DWS), E/A ratio, and mitral valve (MV) deceleration time of mice (*n* = 15). **(J)** Representative echocardiographic images as well as E and A wave Doppler of each group. **(K–L)** Statistical results for the HW/BW and HW/TL (*n* = 15).^∗^*P* < 0.05 versus sham+vehicle, ^#^*P* < 0.05 versus ISO+vehicle.

Geniposide (GE) (**Figure 1A**) is a major component of the fruit of Gardenia jasminoides Ellis (*Gardenia fruits*), which has been experimentally proved to have multiple pharmacological effects, such as anti-inflammation (Koo et al., 2006), anti-apoptosis (Gao et al., 2014), anti-oxidative stress (Lv et al., 2014), anti-angiogenesis (Koo et al., 2004), and anti-ER stress (Cui et al., 2018). Our previous study demonstrated that GE remarkably blocked cardiac hypertrophy through activation of AMPKα and suppression of its downstream mTOR and ERK signaling pathway in the mouse receiving transverse aortic constriction (TAC) (Ma et al., 2016b). However, the effects of GE on cardiac fibrosis and its precise mechanisms remain elusive. Isoproterenol (ISO) is currently used as a research means for the induction of cardiac fibrosis because it could activate the renin–angiotensin system (RAS) and increase the synthesis of collagen (Ocaranza et al., 2002; Zhao et al., 2017). Therefore, this study was designed to clarify the role of GE in cardiac fibrosis induced by ISO and in neonatal rat cardiac fibroblasts stimulated by TGF-β.

## Materials and Methods

### Chemicals and Reagents

Geniposide (≥98% purity, as detected by high-performance liquid chromatography analysis) was purchased from Shanghai Winherb Medical Co. (Shanghai, China). TGF-β (T7039) was obtained from Sigma-Aldrich (St. Louis, MO, United States). ISO was purchased from Sigma-Aldrich Co. EX-527 was purchased from MedchemExpress (Sollentuna, Sweden). Antibodies against the following proteins were purchased from Abcam: TGF-β1 (ab66043), SIRT1 (ab110304), gp91 (ab80508), 4-hydroxynonenal (4HNE) (ab46545), thioredoxin2 (ab26320), α-SMA (ab5694), GRP78 (ab955), XBP-1 (ab37152), TGF beta Receptor I (ab31013), TGF beta Receptor II (ab61213), and acetyl-lysine (ab80178). Antibodies against the following proteins were purchased from Cell Signaling Technology: phosphorylated (P-)Smad3 (8769), total (T-)Smad3 (9513s), and GAPDH (2118). The antibodies against P-PERK (sc-32577) and T-PERK (sc13073) were purchased from Santa Cruz Biotechnology. The antibody against ATF6 (15794-1-AP) was purchased from Proteintech Group. The secondary antibodies used in this study were acquired from LI-COR Biosciences (used at 1:10,000 dilution). The GT VisionTM+Detection System/Mo&Rb reagent for immunohistochemistry was obtained from Gene Technology (Shanghai, China). The Alexa Fluor 488-conjugated goat anti-rabbit secondary antibody for immunofluorescence was purchased from LI-COR Biosciences.

### Animals and Experimental Design

All animal experimental procedures in this study were approved by the Animal Care and Use Committee of Renmin Hospital of Wuhan University and were performed in accordance with the Care and Use of Laboratory Animals published by the US National Institute of Health (Revised 2011). Male C57/B6 mice (8–10 weeks) weighing 25.2 ± 2 g were purchased from the Institute of Laboratory Animal Science, Chinese Academy of Medical Sciences (Beijing, China). The animals were fed regular food and water in a specific-pathogen-free (SPF) environment and maintained under temperature (20–22°C), standard lighting (12:12 h, day–night cycle), and humidity (50–60%) conditions for 7 days before experiments. The mice were randomly divided into four groups (15 per group) for treatment, namely, sham+vehicle, sham+GE, ISO+vehicle, and ISO+GE. For the mouse model of cardiac fibrosis, ISO was injected subcutaneous for 14 days (10 mg/kg for 3 days and 5 mg/kg for 11 days) (Jiang et al., 2017). Beginning on the first day that ISO was injected, GE dissolved in sterile saline was given to the mice intragastrically (09:00 h, at a dose of 50 mg/kg/d) for 2 weeks (GE treatment commenced prior to injection of ISO), and the mice in the sham+vehicle and ISO+vehicle groups were given equal volumes of saline (Ma et al., 2016b). At the end of the 2-week treatment period, the mice were sacrificed by injecting excess sodium pentobarbital after echocardiography and hemodynamic parameters were analyzed. Next, the heart weight (HW), body weight (BW), and tibia length (TL) were measured to obtain the HW/BW and HW/TL ratios. The hearts were collected for further molecular biological and pathological research. To assess the effects of GE on SIRT1 in mice treated with ISO, GE pre-treated mice were injected with EX-527 intraperitoneally (10 mg/kg; vehicle: DMSO) (Wang X. et al., 2015).

### Echocardiography Measurement and Invasive Hemodynamic Pressure–Volume Analysis

To assess the cardiac function of mice, echocardiography was carried out by a Mylab 30CV ultrasound system (Esaote S.P.A, Genoa, Italy) with a 10-MHz linear array ultrasound transducer, as reported (Ma et al., 2017a,b). In detail, mice were anesthetized with continuous 1.5–2% isoflurane inhalation after the precordium was shaved. Then, mice were secured on a warming pad (37°C) in a shallow left lateral position. The parameters, including percentage fractional shortening (FS), percentage ejection fraction (EF), LV end-systolic diameter (LVESd), LV end-diastolic diameter (LVEDd), end-diastolic interventricular septal thickness (IVSd), end-systolic interventricular septal thickness (IVSs), and LV posterior wall thickness (LVPWT) at diastole and systole were recorded after a 2D-guided M-mode trace crossing the posterior and anterior wall of the LV was performed as described previously (Gao et al., 2011). The diastolic wall strain was calculated based on [LVPWT(systole)-LVPWT(diastole)]/LVPWT(systole). To be more specific, the parasternal short axis view of the LV was applied to guide calculations of percentage FS, percentage EF, as well as ventricular volumes and dimensions. Meanwhile, atrial contraction flow peak velocity (A, mm/s) and passive LV filling peak velocity (E, mm/s) were obtained from the images of mitral valve Doppler flow based on the apical four-chamber view. Attention was paid not to produce excessive pressure to the chest to avoid deformation and bradycardia of the heart.

Invasive hemodynamic monitoring was carried out by cardiac catheter connected to a Millar Pressure-Volume System (MPVS-400; Millar Instruments). Briefly, mice were first anesthetized with 1.5% isoflurane inhalation. Subsequently, mice were placed on a warmed surgical platform in supine position, and the right carotid artery was clearly exposed under the operating field. Then, a 1.4-French Millar catheter transducer (Millar, Houston, TX, United States) was inserted from the isolated carotid artery into the LV. All data were recorded and analyzed by PVAN data analysis software (Millar, Houston, TX, United States).

### Histological Examination

The obtained hearts were first fixed with 10% formalin overnight. Subsequently, these hearts were dehydrated and embedded in paraffin. After transverse sectioning into 5-μm sections, the hearts were stained with hematoxylin and eosin (H&E) to measure the cross-sectional area of cardiomyocytes; simultaneously, picrosirius red (PSR) staining was performed to evaluate cardiac fibrosis. All of the sections were observed and photographed using a light microscope and a Nikon PhotoImaging System (Tokyo, Japan) at the magnification of 40× and 200×. The obtained photographs were analyzed using a digital analysis software (Image-Pro Plus 6.0) in a blinded manner, with 200 cells per slide assessed for cardiomyocyte area and more than 60 fields per group evaluated for cardiac fibrosis. The fibrosis content in interstitial area was calculated as the mean ratio of the fibrotic tissue area to the total tissue area of all measurements of the section. As for perivascular fibrosis, the fibrosis content was calculated as the mean ratio of fibrotic area around vessel to the area of vessel.

Immunohistochemical staining for 4-HNE and SIRT1 was performed to assess the level of oxidative stress. After the sections underwent antigen retrieval, they were blocked using 8% goat serum in PBS. Next, they were incubated with the antibody against 4-HNE (Abcam, Cambridge, MA, United States) at 1:100 dilution, at 4°C overnight. Subsequently, the sections were incubated with GT VisionTM+Detection System/Mo&Rb reagent for 60 min for 1 h at room temperature and developed using a peroxide-based substrate diaminobenzidine (DAB) kit (Gene Tech, Shanghai, China). Eventually, these sections were dehydrated and cleared in ethanol and xylene, respectively. The fields of view were taken at the magnification of 200×.

### Neonatal Rat Cardiac Fibroblasts Culture and Treatment

Neonatal rat cardiac fibroblasts were isolated from the new-born Sprague-Dawley rats (1–3-day) as previously reported in the literature (Ma et al., 2016a). The LVs of neonatal rats were minced and digested with collagenase II (50 U ml^-1^) and trypsin (0.1%). Then cells were obtained and plated for 1.5 h at 37°C until the cardiac fibroblasts adhered to the wall of plate. After discarding pre-seeding medium-containing cardiomyocytes and unattached cells, the relatively pure cardiac fibroblasts were collected. Cardiac fibroblasts were then cultured in DMEM/F12 with 10% fetal bovine serum (FBS) at 37°C in a humidified incubator (SANYO MCO-18M) with 5% CO_2_. All neonatal rat cardiac fibroblasts were treated within three passage cultures, but only cardiac fibroblasts prior to the third passage could be used in our study. To guarantee the purity of cardiac fibroblasts, the expression of α-actin and vimentin in the cultured cells to the third generation was detected using immunofluorescence. A negative result of anti-α-actinin and positive result of anti-vimentin were regarded as cardiac fibroblasts. After serum starvation for 24 h and synchronization, cardiac fibroblasts were randomly treated with TGF-β (5 or 10 ng/ml) and different GE concentrations for 24 h. GE was dissolved in PBS and diluted to the desired final concentrations. Cell counting kit 8 (C10227 Countess^®^ automated cell counter, Invitrogen, Carlsbad, CA, United States) was used to investigate the cytotoxic effect of GE on cardiac fibroblasts. SIRT1 expression in cardiac fibroblasts was blocked by EX-527 (10 μM) (Jia et al., 2015).

### Immunofluorescence Staining

To explore the fibrosis conditions, immunofluorescence staining was carried out according to previous research (Wu et al., 2017). In brief, cell coverslips were first fixed with 4% formaldehyde and permeabilized in 0.2% Triton X-100. Then, these coverslips were stained with an antibody against α-SMA (1:100) or SIRT1 (1:100) after blocking with 10% goat serum for 1 h at 37°C. An Alexa Fluor 488-conjugated goat anti-rabbit secondary antibody (1:200) was used for visualization of the coverslips. DAPI was used to counterstain the nuclei in cardiac fibroblasts. Photographs were captured using an OLYMPUS DX51 fluorescence microscope (Tokyo, Japan). The fields of view were taken at the magnification of 200× and 400×. All the photographs obtained were further analyzed in a blinded fashion by Image-Pro Plus 6.0 software.

### Western Blot and Quantitative Real-Time PCR

Protein extraction, SDS–PAGE, and immunodetection of the cardiac tissues and cardiac fibroblasts were performed according to our previous research (Wu et al., 2017). Protein expression levels were normalized to the matched total proteins or GAPDH. Nuclear and cytosolic protein fractions were separated using a commercial kit (Thermo Fisher Scientific) based on the manufacture’s protocol. Intact cell nucleus were isolated from cytoplasm after cardiac fibroblasts were lysed step by step. Then the nuclear protein fractions were carefully extracted from genomic DNA and mRNA. Cytosolic protein fractions were isolated from cytoplasmic lysate. Proteins from nuclear lysates were normalized to PCNA while proteins from cytosolic lysates were normalized to GAPDH.

TRIzol reagent (Invitrogen, Carlsbad, CA, United States) was used to extract total mRNA from tissues or cells. Then, reverse transcription and RT-PCR were performed as reported previously (Ibarra-Lara et al., 2012). GAPDH was used as an internal control. The primers used in this study are shown in **Table 1**.

**Table 1:** Gene-specific primers used in quantitative real-time PCR.

Species	Genes		Sequences
Mouse	GAPDH	Forward	5′-ACTCCACTCACGGCAAATTC-3′
		Reverse	5′-TCTCCATGGTGGTGAAGACA-3′
Mouse	SIRT1	Forward	5′-GACAGAACGTCACACGCCA-3′
		Reverse	5′-ATTGTTCGAGGATCGGTGCC-3′
Mouse	Collagen I	Forward	5′-CCCAACCCAGAGATCCCATT-3′
		Reverse	5′-GAAGCACAGGAGCAGGTGTAGA-3′
Mouse	Collagen III	Forward	5′-GATCAGGCCAGTGGAAATGT-3′
		Reverse	5′-GTGTGTTTCGTGCAACCATC-3′
Mouse	TGF-β1	Forward	5′-ATCCTGTCCAAACTAAGGCTCG-3′
		Reverse	5′-ACCTCTTTAGCATAGTAGTCCGC-3′
Rat	GAPDH	Forward	5′-GACATGCCGCCTGGAGAAAC-3′
		Reverse	5′-AGCCCAGGATGCCCTTTAGT-3′
Rat	SIRT1	Forward	5′-TCTCCCAGATCCTCAAGCCA-3′
		Reverse	5′-CTGCAACCTGCTCCAAGGT-3′
Rat	Collagen I	Forward	5′-TGCTGCCTTTTCTGTTCCTT-3′
		Reverse	5′-AAGGTGCTGGGTAGGGAAGT-3′
Rat	Collagen III	Forward	5′-GTCCACGAGGTGACAAAGGT-3′
		Reverse	5′-CATCTTTTCCAGGAGGTCCA-3′
Rat	Ctgf	Forward	5′-CTAAGACCTGTGGAATGGGC-3′
		Reverse	5′-CTCAAAGATGTCATTGCCCCC-3′

### Determination of SIRT1 Deacetylase Activity

The total proteins extracted from tissues or cells were adjusted to equal concentrations before they were used to detect the deacetylase activity. The reagents from a fluorometric SIRT1 Activity Assay Kit (ab156065, Abcam, Cambridge, MA, United States) were used according to the manufacturer’s protocol. The emission and excitation wavelengths were 450 and 360 nm, respectively. All experimental data are represented as activity compared to that detected in the sham or PBS group.

### Determination of Oxidative Stress

To determine the conditions of oxidative stress of heart tissues, the activity of SOD, MDA, GSH-Px, and thioredoxin reductase were detected using an assay kit (Beyotime, China) according to the standard operational procedure. Similarly, cardiac fibroblasts were lysed and centrifuged to obtain the supernatant fractions, then, the activities of SOD, MDA, GSH-Px, and thioredoxin reductase were detected by a commercially available kit.

### Statistical Analysis

All values are presented as the mean ± SD. Statistical comparisons between two groups were performed using Student’s *t*-test, and comparisons among multiple groups were performed using one-way ANOVA followed by Tukey’s *post hoc* test. All statistical analyses were performed by SPSS 19.0 software. Differences at *P* < 0.05 were considered statistically significant.

## Results

### GE Significantly Improved Cardiac Function in Mice Challenged With ISO

Consistent with our previous research, mice treated with ISO exhibited obviously worse cardiac function (Jiang et al., 2017). No significant difference in heart rate among these groups was observed (**Figure 1B**). The levels of EF, FS, the interventricular septal thickness at systole or diastole (IVSs or IVSd) in mice challenged with ISO were significantly reversed by GE administration (*P*<0.05) (**Figures 1C–F**). The diastolic wall strain, E/A ratio, and the mitral valve deceleration time nearly went back to the normal level in the ISO+GE group, indicating that GE treatment improved the diastolic function in the ISO-induced mice (**Figures 1G–I**). Additionally, echocardiographic images and hemodynamic parameters also showed that GE conferred better systolic and diastolic left ventricular functions in the mice treated with ISO compared with those without GE administration, as assessed by ESP, EDP, d*p*/d*t* min, and d*p*/d*t* max (**Figure 1J** and **Table 2**). Also, the level of HW/TL and HW/BW was also significantly reversed by GE administration (**Figures 1K–L**). Taken together, these data suggested that GE administration could lead to alleviated cardiac dysfunction in mice challenged with ISO.

**Table 2:** Hemodynamic parameters in mice after 2 weeks of ISO injection.

Parameter	Sham		ISO	
	Vehicle (*n* = 6)	GE (*n* = 6)	Vehicle (*n* = 6)	GE (*n* = 6)
HR (bpm)	469.4 ± 7.8	475.1 ± 12.3	471 ± 10.1	466 ± 11.2
LVESP (mmHg)	109.1 ± 5.6	108.4 ± 6.8	148.1 ± 11.3^∗^	136.2 ± 10.8^#^
LVEDP (mmHg)	10.6 ± 2.4	11.8 ± 5.9	16.8 ± 2.9^∗^	13.5 ± 1.6^#^
d*p*/d*t* min (mmHg/s)	-8456.9 ± 433.9	-8823.8 ± 246.6	-5012 ± 544.9^∗^	-6914 ± 631.4^#^
d*p*/d*t* max (mmHg/s)	9677.1 ± 512.1	9100.2 ± 435.0	6120.5 ± 611.2^∗^	7600.9 ± 213.9^#^

*HR, heart rate; LVESP, left ventricular end-systolic pressure; LVEDP, left ventricular end-diastolic volume; d*p*/d*t* min, minimal rate of pressure decay; d*p*/d*t* max, maximal rate of pressure development. All values are presented as the mean ±*SD*. *^∗^P* < 0.05 compared with the Sham group, ^#^*P* < 0.05 compared with the ISO+Vehicle group*.

### GE Alleviated Cardiac Fibrosis Induced by ISO *in vivo*

**FIGURE 2:**
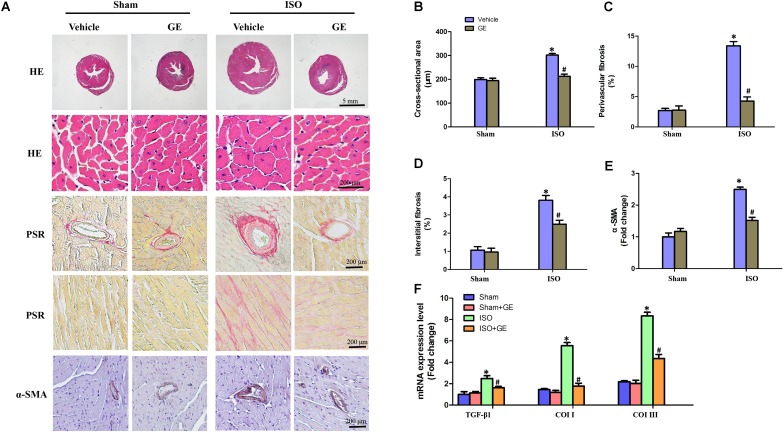
Effect of GE treatment on ISO-induced cardiac fibrosis in mice. **(A)** Representative images of the morphological analysis of cardiac fibrosis as reflected by the H&E staining, PSR staining, and immunohistochemistry staining for α-SMA protein. **(B–D)** Statistical results for the cardiomyocyte cross-sectional area and percentage fibrosis of perivascular as well as interstitial fibrosis (*n* = 6). **(E)** Quantitative results of α-SMA of immunohistochemistry staining showing the activation of myofibroblasts. **(F)** The relative mRNA levels of TGF-β1, collagen I (col I), and collagen III (col III) normalized to Gapdh in mice (*n* = 6). ^∗^*P* < 0.05 versus sham+vehicle, ^#^*P* < 0.05 versus ISO+vehicle.

Cardiac fibrosis is one of the main features in the progression of cardiac remodeling, which eventually contributes to heart failure by increasing ventricular stiffness and decreasing pumping capability (Conrad et al., 1995). Here, we explored the effect of GE on cardiac remodeling. As shown in **Figures 2A–D**, H&E staining showed that GE-treated mice significantly reversed the ISO insult-induced morphological heart alteration, as evidenced by a smaller myocyte cross-sectional area. PSR-stained images demonstrated that GE-treated mice exhibited a much lower quantification of percentage fibrosis in both interstitial and perivascular area, which was verified by the suppressed mRNA levels of the fibrosis markers TGF-β1, Col I, and Col III (**Figure 2F**). Also, GE treatment reduced the expression of α-SMA in ISO-induced hearts (**Figures 2A,E**).

### GE Promoted SIRT1 Activation and Blocked Oxidative Stress, ER Stress, and the TGF-β1/Smad3 Pathway *in vivo*

**FIGURE 3:**
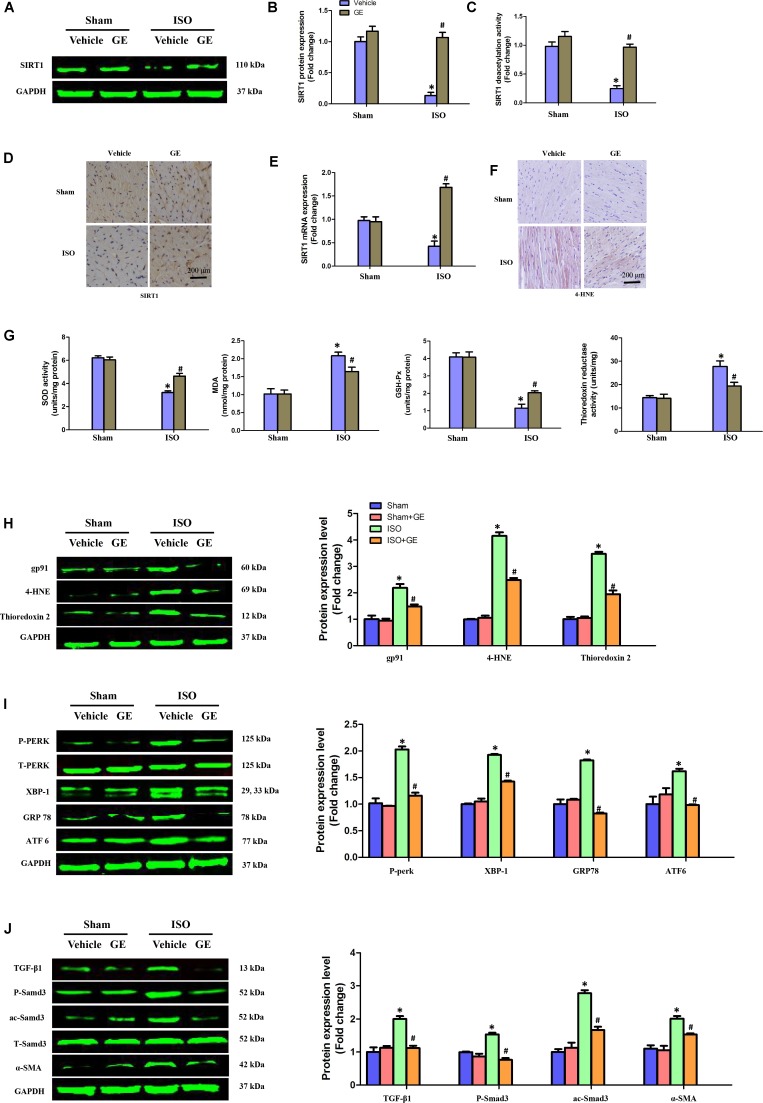
GE suppressed oxidative stress, endoplasmic reticulum (ER) stress, and TGF-β1/Smad3 signaling pathway in mice induced by ISO. **(A,B)** Western blot and quantification of SIRT1 protein expression levels normalized to GAPDH in hearts of each group (*n* = 6). **(C)** Relative SIRT1 deacetylation activity (*n* = 6). **(D)** Representative images of immunohistochemical staining for the SIRT1 protein. **(E)** The relative mRNA level of SIRT1 in heart normalized to Gapdh in mice (*n* = 6). **(F)** Representative images of immunohistochemical staining for the 4-HNE protein. **(G)** The SOD activity, malondialdehyde (MDA) production, GSH-Px activity, and thioredoxin reductase activity in the indicated group (*n* = 6). **(H–J)** Western blotting and quantitative analysis of oxidative stress markers (gp91, 4-HNE, and thioredoxin2 normalized to GAPDH), ER stress markers (P-PERK normalized to T-PERK, XBP-1, GRP78, and ATF6 normalized to GAPDH), P-Smad3, and ac-Smad3 normalized to T-Smad3, TGF-β1, and α-SMA normalized to GAPDH (*n* = 6). ^∗^*P* < 0.05 versus sham+vehicle, ^#^*P* < 0.05 versus ISO+vehicle.

Accumulating evidence has shown that SIRT1 may exert favorable cardiovascular protective effects by regulating oxidative stress, ER stress, and the TGF-β1/Smad3 pathway, which are crucial for the development of cardiac fibrosis (Alcendor et al., 2007; Cappetta et al., 2015; Guo et al., 2015). Our results showed that ISO obviously suppressed SIRT1 expression at the mRNA and protein levels, in addition to reducing deacetylation activity. However, the inhibitory effect of ISO on SIRT1 was counteracted by GE administration (50 mg/kg/d) (**Figures 3A–E**). To further explore the precise mechanisms contributing to the protective effect of GE, we subsequently measured the levels of protein markers associated with oxidative stress, ER stress, and the Smad3 pathway. Immunohistochemical staining and Western blot revealed that the production of 4-HNE, a typical oxidative product, in mouse heart induced by ISO was significantly reversed by GE (**Figure 3F**). Meanwhile, GE significantly improved antioxidative enzyme activities and suppressed some typical oxidative enzyme activities to prevent heart from ISO injuries, as indicated by the increased SOD and GSH-Px activity and the reduced MDA levels and thioredoxin reductase activity (**Figure 3G**). Meanwhile, the levels of gp91, 4-HNE, and thioredoxin 2 were significantly reversed by GE (**Figure 3H**). Additionally, GE inhibited ER stress in ISO-induced hearts, as demonstrated by decreased phosphorylation of protein kinase dsRNA-dependent-like ER kinase (PERK) and reduced expression of glucose-regulated protein 78 (GRP78), X-box binding protein 1 (XBP-1), and activating transcription factor 6 (ATF6), which are all classical markers of ER stress (**Figure 3I**). Moreover, the activation of the ac-Smad3 and P-Smad3 pathways in ISO-induced heart was also suppressed (**Figure 3J**). These data suggested that GE attenuated cardiac fibrosis via inhibition of oxidative stress, ER stress, and Smad3 phosphorylation and acetylation, in addition to SIRT1 activation.

### GE Prevented the Accumulation of Collagen in Cardiac Fibroblasts Stimulated by TGF-β1 Partially in a SIRT1-Dependent Manner *in vitro*

**FIGURE 4:**
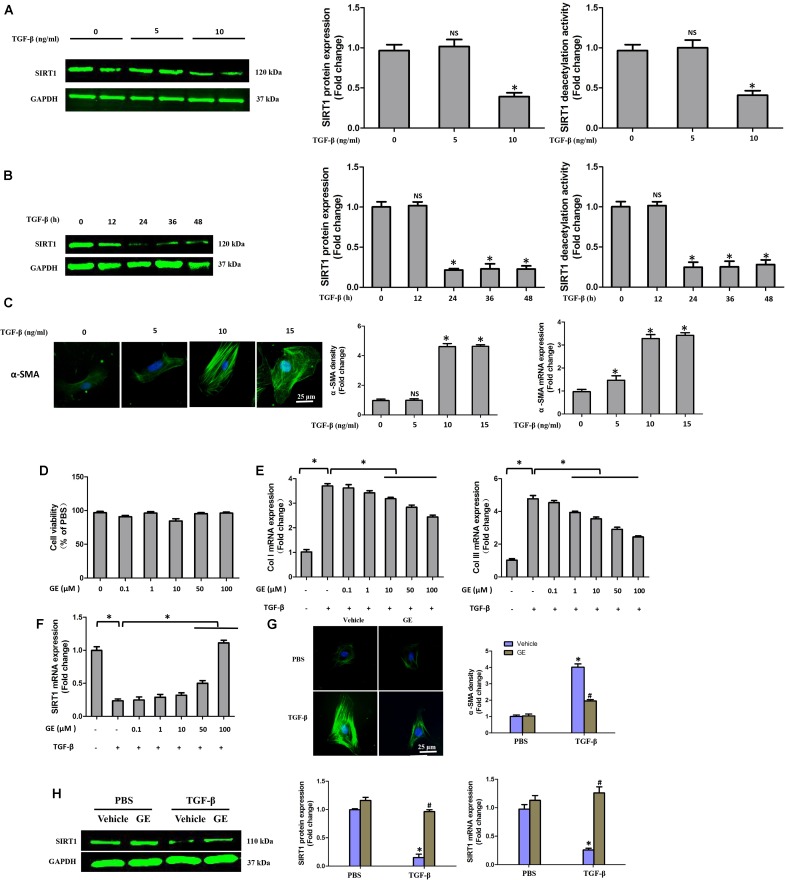
GE alleviated collagen synthesis of cardiac fibroblasts stimulated by TGF-β. **(A)** SIRT1 protein expression level and deacetylation activity in cardiac fibroblasts stimulated by different doses of TGF-β (0, 5, and 10 ng/ml) for 24 h. **(B)** SIRT1 protein expression level and deacetylation activity in cardiac fibroblasts stimulated by TGF-β (10 ng/ml) for different times (0, 12, 24, 36, and 48 h). **(C)** Representative images of immunofluorescence staining of α-SMA as well as its mRNA expression levels normalized to gapdh in cardiac fibroblasts stimulated by different doses of TGF-β (0, 5, and 10 ng/ml) for 24 h. **(D)** Cytotoxicity of GE on cardiac fibroblasts was assessed by CKK-8 assay (*n* = 6). **(E)** The relative mRNA levels of col I and col III normalized to Gapdh in cardiac fibroblasts that were co-incubated with TGF-β (10 ng/ml) and GE (0, 0.1, 1, 10, 50, or 100 μM) for 24 h (*n* = 3). **(F)** The relative mRNA level of SIRT1 in cardiac fibroblasts (*n* = 3). **(G)** Representative images of immunofluorescence staining of α-SMA in fibroblasts stimulated by TGF-β (10 ng/ml) for 24 h after GE treatment (100 μM). **(H)** The protein expression and mRNA expression of SIRT1 in cardiac fibroblasts stimulated by TGF-β (10 ng/ml) for 24 h after GE treatment (100 μM) (*n* = 6).

**FIGURE 5:**
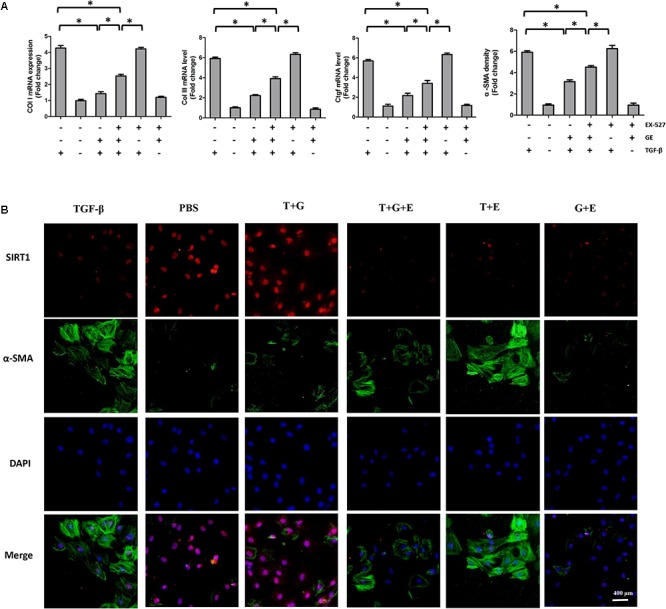
EX-527 partially counteracted the inhibitory effects of GE on collagen synthesis and transformation of cardiac fibroblasts to myofibroblasts. **(A)** The relative mRNA levels of col I, col III, Ctgf, and α-SMA normalized to Gapdh under TGF-β stimulation with or without GE and EX-527 (*n* = 3). **(B)** Representative images of immunofluorescence staining of α-SMA and SIRT1. Green fluorescence indicates α-SMA, red fluorescence indicates SIRT1, and nuclei were labeled using DAPI (blue). T stands for TGF-β1, G stands for GE, and E stands for Ex-527. ^∗^*P* < 0.05 versus the matched group.

**FIGURE 6:**
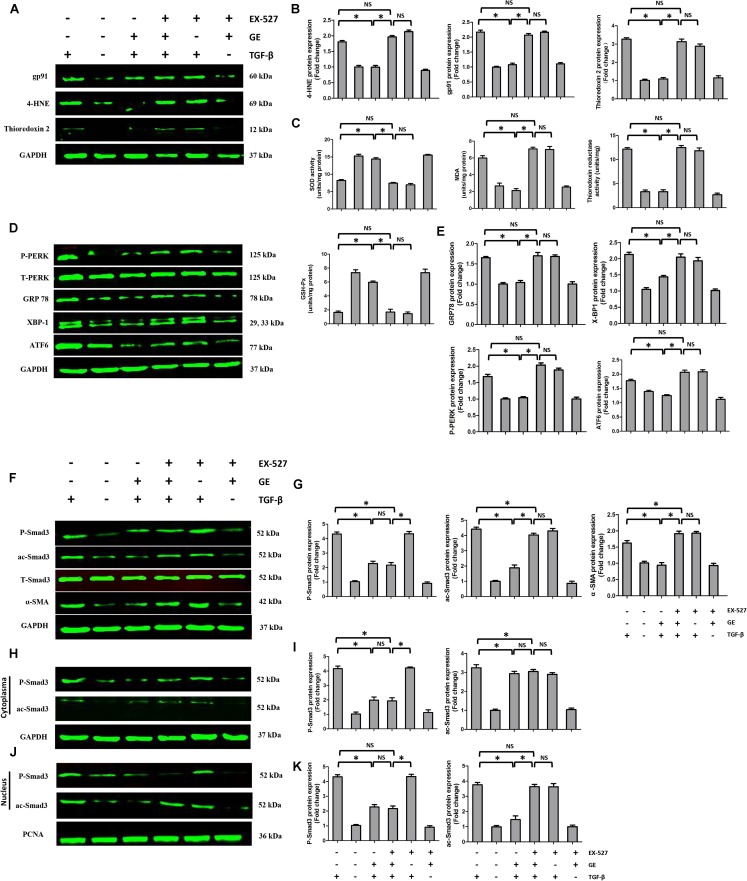
Effects of GE on oxidative stress, ER stress, and Smad3 pathway in cardiac fibroblasts. **(A,B)** Western blotting and quantitative analysis of oxidative stress marker levels including gp91, 4-HNE, and thioredoxin 2 normalized to GAPDH (*n* = 6). **(C)** SOD activity, MDA production, thioredoxin reductase activity, and GSH-Px activity in the indicated groups (*n* = 6). **(D,E)** Western blotting and quantitative analysis of ER stress marker levels including P-PERK normalized to T-PERK, GRP 78, XBP-1, and ATF6 normalized to GAPDH (*n* = 6). **(F,G)** Western blotting and quantitative analysis of cellular P-Smad3 and ac-Smad3 levels normalized to T-Smad3, and α-SMA levels normalized to Gapdh (*n* = 6). **(H,I)** Western blotting and quantitative analysis of P-Smad3 and ac-Smad3 levels normalized to GAPDH in the cytoplasm (*n* = 6). **(J,K)** Western blotting and quantitative analysis of P-Smad3 and ac-Smad3 levels normalized to PCNA in the nucleus (*n* = 6). ^∗^*P* < 0.05 versus the matched group. NS indicates no significant difference compared with the matched group.

**FIGURE 7:**
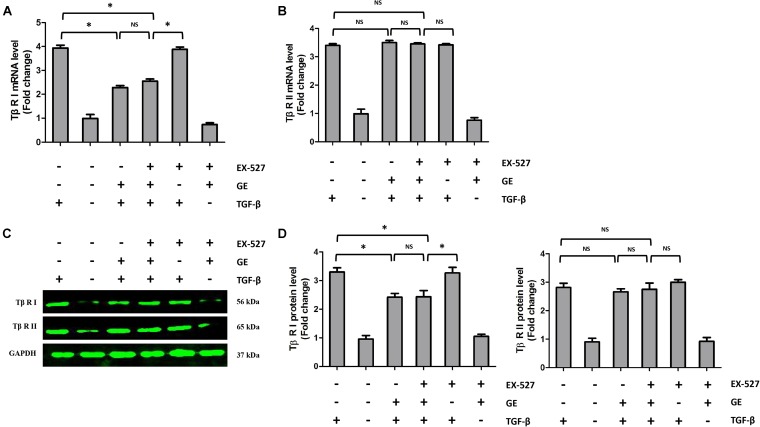
Effects of GE on Tβ-RI and Tβ-RII in cardiac fibroblasts. **(A,B)** The relative mRNA levels of Tβ-RI and Tβ-RII normalized to Gapdh in cardiac fibroblasts (*n* = 6). **(C,D)** Western blotting and quantitative analysis of Tβ-RI and Tβ-RII levels normalized to GAPDH in cardiac fibroblasts (*n* = 6). ^∗^*P* < 0.05 versus the matched group. NS indicates no significant difference compared with the matched group.

It is well recognized that cardiac fibroblasts are the primary cell type contributing to cardiac fibrosis in cardiac remodeling. After heart injury, activated cardiac fibroblasts from all sources begin to proliferate and transdifferentiate to the myofibroblasts, starting to secrete collagens and other ECM proteins (Fan and Guan, 2016). Therefore, we further explored the pharmacological effects of GE as well as the mechanisms by which GE acts on cardiac fibroblasts stimulated by TGF-β *in vitro*. SIRT1 protein expression level and deacetylation activity were significantly decreased by TGF-β at a concentration of 10 ng/ml (**Figure 4A**). Therefore, we selected 10 ng/ml TGF-β to stimulate cardiac fibroblasts for 0, 12, 24, 36, and 48 h and found that both the expression level and deacetylation activity of SIRT1 were clearly decreased at 24, 36, and 48 h (**Figure 4B**). As shown in **Figure 4C**, the level of α-SMA significantly increased when the TGF-β concentration was over 10 ng/ml. Based on these results, in the following experiments, cardiac fibroblasts were incubated with 10 ng/ml TGF-β for 24 h. Because the anti-fibrotic effect mediated by GE may cause cell damage activity, we measured the viability of cardiac fibroblasts exposed to different concentrations of GE (0–100 μM), and no cytotoxicity was observed (**Figure 4D**). Subsequently, we applied different GE concentrations to cardiac fibroblasts stimulated by TGF-β1. Only the GE concentrations of 10, 50, and 100 μM displayed an obvious inhibitory effect in a dose-dependent fashion on both collagen I and collagen III mRNA levels in cultured neonatal cardiac fibroblasts stimulated by TGF-β (**Figure 4E**). However, only the 50 and 100 μM GE concentrations enhanced SIRT1 expression (**Figure 4F**). Hence, 100 μM GE was selected for further study. As expected, GE (100 μM, 12 h) significantly reduced the α-SMA density in cardiac fibroblasts stimulated by TGF-β (**Figure 4G**), accompanied by an increase in SIRT1 expression at both the protein and mRNA levels (**Figure 4H**). To further investigate whether the protective effects of GE depended on SIRT1 activation, we used EX-527 to inhibit SIRT1 activation in cardiac fibroblasts. Intriguingly, the protective effects of GE on cardiac fibroblasts stimulated by TGF-β were incompletely blocked (**Figures 5A–B**), accompanied by the activation of oxidative stress, ER stress, and the ac-Smad3 pathway (**Figures 6A–E**). However, P-Smad3 protein expression in both the cytoplasm and the nucleus was still inhibited by GE regardless of SIRT1 inactivation, indicating that SIRT1 may have no effects on Smad3 phosphorylation (**Figures 6F–K**). To find the reason for the decrease in P-Smad3 levels, we determined the mRNA levels of Tβ RI and Tβ RII, which are directly responsible for the phosphorylation of Smad3. As shown in **Figures 7A–D**, GE could significantly decrease the expression of Tβ RI at the protein and mRNA level independent of SIRT1 inactivation. The above data suggested that GE could attenuate TGF-β-induced ECM production in cardiac fibroblasts partially in a SIRT1-dependent manner, and the underlying mechanism may be associated with the inhibitory effects of GE on oxidative stress, ER stress, and the ac-Smad pathway. Additionally, GE prevented ECM production by reducing the expression of Tβ RI, thereby inhibiting Smad3 phosphorylation.

### GE Exhibited Weakened Effects After SIRT1 Was Blocked in Mice

**FIGURE 8:**
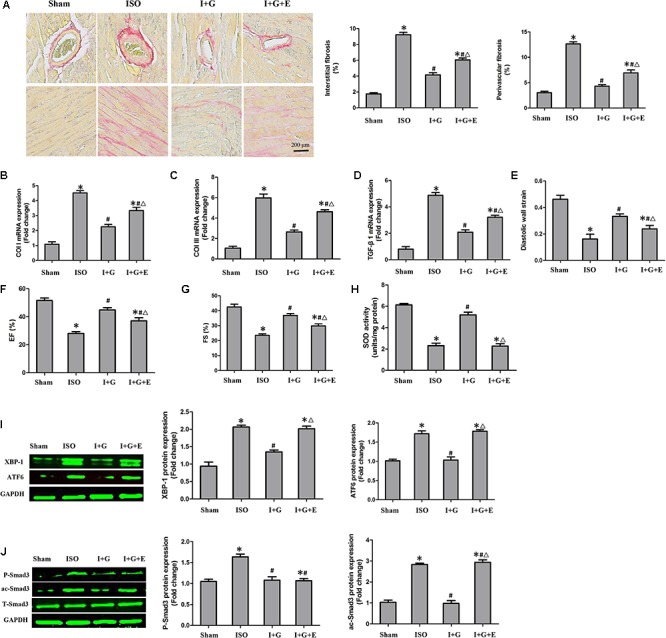
The cardioprotective and anti-fibrotic protective effect of GE was partially prevented following SIRT1 inhibition. **(A)** Representative images of PSR staining and quantitative results of percentage fibrosis of perivascular and interstitial fibrosis. **(B–D)** The relative mRNA levels of col I, col III, and TGF-β1 normalized to Gapdh in mice (*n* = 6). **(E–G)** Echocardiographic parameters including DWS, EF, and FS (*n* = 6). **(H)** The SOD activity in the indicated group (*n* = 6). **(I)** Western blotting and quantitative analysis of XBP-1 and ATF6 levels normalized to GAPDH in mice (*n* = 6). **(J)** Western blotting and quantitative analysis of P-Smad3 and ac-Smad3 levels normalized to T-Smad3 (*n* = 6). I stands for ISO, G stands for GE, and E stands for Ex-527. ^∗^*P* < 0.05 versus the sham+vehicle group, ^#^*P* < 0.05 versus the ISO+vehicle group, and ^Δ^*P* < 0.05 versus the I+G group.

Next, we investigated whether SIRT1 was essential for the protective effects of GE *in vivo*. After treatment with EX-527, the anti-fibrotic effect of GE in the heart induced by ISO was significantly weakened but not abolished, as evidenced by the increased LV percentage fibrosis in perivascular and interstitial regions as well as the up-regulated mRNA expression of collagen I, collagen III, and TGF-β1 (**Figures 8A–D**). Additionally, cardiac function in mice treated with EX-527 was also decreased but to a lesser extent than in the ISO group, despite GE administration (**Figures 8E–G**). We also examined the SOD activity and P-Smad3, ac-Smad3, XBP-1, and ATF6 expression levels. As expected, after EX-527 was applied, GE treatment did not alter SOD activity (**Figure 8H**) or the protein levels of ac-Smad3, XBP-1, and ATF6 in mice treated with ISO (**Figure 8I**). However, the level of P-Smad3 in the I+G+E group was still at a low level relative to that in the ISO+GE group (**Figure 8J**). These results further suggested that GE protects against cardiac fibrosis by inhibiting oxidative stress, ER stress, and the ac-Smad3 pathway in a SIRT1-dependent manner and suppressing Smad3 phosphorylation independently of SIRT1 activation.

## Discussion

**FIGURE 9:**
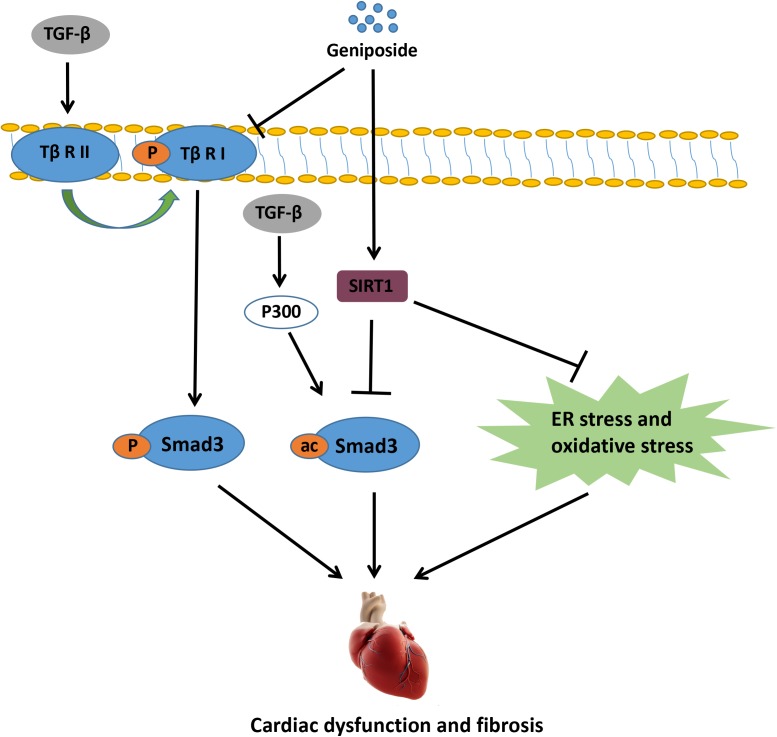
The proposed mechanisms for the protective role of GE in ISO-induced cardiac fibrosis.

In the present study, we showed that GE mitigated ISO-induced cardiac dysfunction and fibrosis *in vivo*. Additionally, GE treatment blocked the transformation of cardiac fibroblasts into myofibroblasts *in vitro*. To the best of our knowledge, our study is the first to prove that SIRT1 is dramatically decreased in cardiac fibrosis induced by ISO or cardiac fibroblasts stimulated by TGF-β, and GE may protect against cardiac fibrosis partially via SIRT1 activation. The activated SIRT1 suppressed the activation of Smad3 acetylation, oxidative stress, and ER stress, thereby preventing cardiac fibrosis. Additionally, GE may significantly inhibit the phosphorylation of Smad3 by reducing the level of Tβ RI but not Tβ RII independently of SIRT1 activation (**Figure 9**).

Geniposide is one of the natural components extracted from plants, which has been traditionally used as a folk medicine for hundreds of years in Asian countries. Until now, GE has been extensively used for the reason that it rarely causes severe toxic effects (Sato et al., 2007), while possessing various pharmacological properties involving anti-inflammatory (Wang et al., 2012), anti-apoptosis (Jiang et al., 2016), anti-thrombus (Zhang et al., 2013), anti-oxidative stress (Wang J. et al., 2015), and anti-ER stress (Lee et al., 2013) effects. In our previous study, GE was shown to retard cardiac hypertrophic response and activate AMPK in the hearts of mice subjected to TAC (Ma et al., 2016b). Particularly, in hepatic fibrosis, GE exerted significant protective effects by preventing TGF-β-induced epithelial–mesenchymal transition (EMT) via inhibition of the TGFβ/Smad and ERK signaling pathways (Park et al., 2015). However, the GE role in cardiac fibrosis has not been completely elucidated.

Sirtuin 1 deacetylase is a class III NAD^+^-dependent deacetylase, which could deacetylate various proteins in cells, involving DNA repair proteins, autophagy factors, transcription factors, and histones (Eaton, 2001). Emerging evidence has proved that SIRT1 serves as a vital therapeutic target for preventing various fibrotic diseases (Huang et al., 2013; Simic et al., 2013; Cappetta et al., 2015). For instance, knockdown of SIRT1 promoted the TGF-β1-mediated fibroblast differentiation and activation in lung by the mechanism that implied the regulation of expression of P300 (Zeng et al., 2017). Additionally, in patients with chronic kidney disease, SIRT1 activation could protect against renal fibrosis by inhibiting the TGF-β/Smad3 pathway (Huang et al., 2014). In the heart, SIRT1 expression was significantly decreased in a model of anthracycline cardiomyopathy, but SIRT1 activation could reverse diastolic dysfunction and alleviate cardiac fibrosis by abolishing the pro-fibrotic TGF-β/SMAD3 pathway (Cappetta et al., 2015). Based on these studies, we detected SIRT1 expression in mouse heart challenged with ISO and cardiac fibroblasts stimulated by TGF-β. As expected, SIRT1 expression both *in vivo* and *in vitro* was dramatically decreased. However, GE treatment inhibited SIRT1 inactivation and significantly attenuated cardiac dysfunction and cardiac fibrosis. Mountainous evidence has disclosed that ISO treatment could up-regulate the expression of TGF-β in the model of cardiac fibrosis, in addition to previous and our present study (Jiang et al., 2017; Li M. et al., 2013; Li et al., 2017; Zhang W. et al., 2017). Meanwhile, the stimulation of TGF-β significantly decreased the expression of SIRT1 in cardiac fibroblasts (Zhang J. et al., 2017). Hence, we speculate that the mechanism of down-regulation of SIRT1 expression induced by ISO may be explained by the increased TGF-β.

Endoplasmic reticulum stress is usually caused by the accumulation of unfolded proteins induced by various stresses, which imbalance the redox state, the cellular energy levels, and the Ca^2+^ concentration, giving rise to activation of the UPR pathway (Volmer et al., 2013). The UPR initially could maintain the normal ER function by increasing the production of chaperone GRP78, subsequently activating the UPR signaling molecules inositol-requiring 1α (IRE1α), PERK, and ATF6 (Lee et al., 2014). Accumulating evidence has demonstrated a prominent role of ER stress and UPR activation in fibrotic conditions, which affect many internal organs, including liver, lung, heart, and kidney. In the model of cardiac fibrosis induced by ISO, *in vivo* administration of 4-PBA, a chemical chaperone enhancing the ER luminal folding capacity, inhibited activation of ER stress, collagen deposition, and cardiac fibrosis, suggesting that inhibiting ER stress may be a potent approach to treating cardiac fibrosis (Ayala et al., 2012). Guo et al. (2015) reported that SIRT1 activation could reduce diabetic cardiomyopathy-induced cardiomyocyte apoptosis by affecting ER stress. Additionally, in the mouse model of renal fibrosis, SIRT1 upregulation could inhibit the ER stress through induction of heme oxygenase-1 (HO-1) and thioredoxin (Chang et al., 2016). On the other hand, oxidative stress is also a key pathophysiological process in the development of cardiac fibrosis. Previous studies have demonstrated that oxidative stress in the heart could mediate collagen synthesis and block collagen degradation in hypertensive rats and diabetic rats (Aragno et al., 2008; Zhao et al., 2008). Consistent with these studies, we showed that GE could suppress ER stress and oxidative stress in a SIRT1-dependent manner. Intriguingly, Alcendor et al. (2007) reported that mild-to-moderate (2.5- to 7.5-fold) overexpression of SIRT1 could be anti-aging and protect against oxidative stress associated with cardiac hypertrophy and fibrosis, however, higher SIRT1 levels, on the contrary, could contribute to cardiomyopathy by promoting mitochondrial dysfunction. Here, although activated SIRT1 may be considered as a promising therapy for cardiac fibrosis, careful assessment regarding the doses is necessary to exert the therapeutic potential of SIRT1.

However, how SIRT1 activation by GE decreases the expression of ac-Smad3 and oxidative stress and ER stress marker genes remains unclear. The activation of AMP-activated protein kinase (AMPK) is one of the core sensors of cellular energy status, serving as a downstream factor of SIRT1. In heart failure, resveratrol, a potent SIRT1 activator, could increase AMPK expression and alleviate cardiac dysfunction through SIRT1 activation (Gu et al., 2014). Additionally, swimming training prevented ISO-induced cardiac fibrosis through the blockage of the AMPK-mediated NADPH oxidase–ROS pathway (Zhao et al., 2008). We hypothesize that the inhibitory effects of GE-activated SIRT1 on Smad3 acetylation, oxidative stress, and ER stress are mediated by AMPK. However, this hypothesis will require adequate studies to draw convincing conclusions.

Apart from inhibiting Smad3 acetylation in a SIRT1-dependent manner, GE had a significant inhibitory effect on the phosphorylation of Smad3 despite the suppression of SIRT1 activity, indicating that GE decreased Smad3 phosphorylation independently of SIRT1 activation. Traditionally, Smad3 phosphorylation is regarded as one of the main post-transcriptional modifications in protein epigenetics contributing to fibrosis because Smad3 but not Smad2 possesses a transcriptional activator domain and DNA-binding activity, which could direct binding to target genes (Roberts et al., 2006). In the fibrotic heart, TGF-β1 could interact with Tβ RII and form a heterodimer to phosphorylate Tβ RI. Then, Smad3 could be directly phosphorylated by Tβ RI and enter the nucleus to trigger a fibrotic response (Bujak et al., 2007). In our study, GE significantly decreased the protein level of P-Smad3 in both the cytoplasm and the nucleus, regardless of SIRT1 inhibition. However, GE had no obvious effect on the protein level of ac-Smad3 in the cytoplasm, regardless of SIRT1 inhibition. Intriguingly, the level of ac-Smad3 in the nucleus was significantly reduced by GE, but SIRT1 inhibition abolished this effect. This result may be explained by the location of SIRT1 in the cell. SIRT1, as a deacetylase, mainly exists in the nucleus (Lin and Fang, 2013); thus, it primarily affects ac-Smad3 in the nucleus, whereas it does not affect cytoplasmic ac-Smad3 or P-smad3. To further explore this mechanism, we detected the mRNA levels of Tβ RI and Tβ RII in cardiac fibroblasts. GE did not affect the mRNA level of Tβ RII in cardiac fibroblasts stimulated by TGF-β despite inhibition of SIRT1. Intriguingly, the mRNA level of Tβ RI was significantly reduced by GE independently of SIRT1 inhibition. Indeed, after SIRT1 was blocked, the phenotype in both mice and cells was obviously worse than that in the group not treated with the SIRT1 inhibitor, although the protective effect of GE still existed compared with the disease group. These data showed that SIRT1 activation was not the only reason for the anti-fibrotic effect of GE.

## Conclusion

Our study indicates that GE may attenuate ISO-induced cardiac dysfunction and fibrosis as well as transformation of cardiac fibroblasts into myofibroblasts upon stimulation by TGF-β partly in a SIRT1-dependent manner, the mechanism of which is associated with the suppression of the ac-Smad3 pathway, oxidative stress, and ER stress. Additionally, GE may decrease P-Smad3 levels by reducing the level of Tβ RII independent of SIRT1 activation. Thus, activation of SIRT1 by GE in the heart may be a promising therapeutic strategy against cardiac fibrosis. However, further investigation aiming to elucidate the potential value of GE in different animal models is of vital importance.

## Author Contributions

NL, HZ and Z-GM designed and performed the experiment. J-XZ, PS and CL carried out the experiment. CY-K and H-MW analyzed the data and contributed to the writing. WD and Q-ZT were in charge of the project and responsible for writing.

## Conflict of Interest Statement

The authors declare that the research was conducted in the absence of any commercial or financial relationships that could be construed as a potential conflict of interest.
